# Author Correction: Effective screening strategies for safe opening of universities under Omicron and Delta variants of COVID-19

**DOI:** 10.1038/s41598-022-27068-z

**Published:** 2022-12-27

**Authors:** Marie Jeanne Rabil, Sait Tunc, Douglas R. Bish, Ebru K. Bish

**Affiliations:** 1 Grado Department of Industrial and Systems Engineering, Virginia Polytechnic Institute and State University, Blacksburg, 24061 USA; 2 Department of Information Systems, Statistics, and Management Science, Culverhouse College of Business, The University of Alabama, Tuscaloosa, 35487 USA

Correction to:* Scientific Reports* 10.1038/s41598-022-25801-2, published on 9 December 2022

The original version of this Article omitted the Funding section. The Funding section now reads:

“We thank VT’s OASF for supporting us in publishing this article.”

The original Article has been corrected.

